# Genetic Variation in *SULF2* Is Associated with Postprandial Clearance of Triglyceride-Rich Remnant Particles and Triglyceride Levels in Healthy Subjects

**DOI:** 10.1371/journal.pone.0079473

**Published:** 2013-11-20

**Authors:** Niina Matikainen, Maria Antonella Burza, Stefano Romeo, Antti Hakkarainen, Martin Adiels, Lasse Folkersen, Per Eriksson, Nina Lundbom, Ewa Ehrenborg, Marju Orho-Melander, Marja-Riitta Taskinen, Jan Borén

**Affiliations:** 1 HUCH Heart and Lung Centre, Cardiovascular Research Group, Helsinki University Central Hospital, Diabetes & Obesity, University of Helsinki, Helsinki, Finland; 2 Department of Molecular and Clinical Medicine/Wallenberg Laboratory, University of Gothenburg, Gothenburg, Sweden; 3 Medical Imaging Center, Helsinki University Central Hospital, Department of Radiology, University of Helsinki, Helsinki, Finland; 4 Atherosclerosis Research Unit, Department of Medicine, Center for Molecular Medicine, Karolinska Institute, Stockholm, Sweden; 5 Department of Clinical Sciences, Lund University, Malmö, Sweden; Vanderbilt University, United States of America

## Abstract

**Context:**

Nonfasting (postprandial) triglyceride concentrations have emerged as a clinically significant cardiovascular disease risk factor that results from accumulation of remnant triglyceride-rich lipoproteins (TRLs) in the circulation. The remnant TRLs are cleared from the circulation by hepatic uptake, but the specific mechanisms involved are unclear. The syndecan-1 heparan sulfate proteoglycan (HSPG) pathway is important for the hepatic clearance of remnant TRLs in mice, but its relevance in humans is unclear.

**Objective:**

We sought to determine whether polymorphisms of the genes responsible for HSPG assembly and disassembly contribute to atherogenic dyslipoproteinemias in humans.

**Patients And Design:**

We performed an oral fat load in 68 healthy subjects. Lipoproteins (chylomicrons and very low density lipoproteins 1 and 2) were isolated from blood, and the area under curve and incremental area under curve for postprandial variables were calculated. Single nucleotide polymorphisms in genes encoding syndecan-1 and enzymes involved in the synthesis or degradation of HSPG were genotyped in the study subjects.

**Results:**

Our results indicate that the genetic variation rs2281279 in *SULF2* associates with postprandial clearance of remnant TRLs and triglyceride levels in healthy subjects. Furthermore, the SNP rs2281279 in *SULF2* associates with hepatic *SULF2* mRNA levels.

**Conclusions:**

In humans, mild but clinically relevant postprandial hyperlipidemia due to reduced hepatic clearance of remnant TRLs may result from genetic polymorphisms that affect hepatic HSPG.

## Introduction

Nonfasting (postprandial) triglyceride (TG) concentrations are a clinically significant risk factor for cardiovascular disease (CVD) [1-3]. TGs are carried in chylomicrons (CMs) synthesized in the intestine and in liver-derived very low density lipoprotein (VLDL). Lipolysis of these triglyceride-rich lipoproteins (TRLs) by lipoprotein lipase (LPL) results in the formation of smaller remnants particles that are depleted of triglycerides and enriched in cholesteryl esters [4]. For many years, CMs and CM remnants were thought to be the major culprits in postprandial hyperlipidemia [5,6]. However, the major increase in the postprandial lipoproteins after food intake occurs in the hepatically derived VLDL remnant particles [7,8]. 

A key step in atherogenesis is entrapment of lipoproteins in the arterial intima [9-11]. Because of their size, most remnant particles cannot cross the endothelium as efficiently as smaller low density lipoprotein (LDL) particles [12]. However, since each remnant particle contain more cholesteryl esters per particle than do LDL [12], elevated levels of remnants may lead to accelerated atherosclerosis and CVD.

The mechanism for delayed postprandial hyperlipidemia involves decreased hepatic clearance of TRL remnants, which is mediated by the syndecan-1 heparan sulfate proteoglycan (HSPG) and the LDL receptor [13-18]. The importance of the HSPG pathway has been questioned, but recent results demonstrate that dysfunction of syndecan-1 HSPG in animal models disrupts defective hepatic remnant clearance [13]. Furthermore, in mice with type 2 diabetes, uptake of remnant lipoproteins is suppressed by accelerated degradation of HSPG owing to the hepatic induction of a heparan sulfate 6-O-endosulfatase (SULF2) [19,20].

These findings raise a clinically relevant question: Do polymorphisms of genes responsible for HSPG assembly and disassembly contribute to atherogenic dyslipoproteinemias in humans? To answer this question, we studied healthy subjects after an oral fat load. Our results indicate association between a genetic variant of *SULF2* and disturbed hepatic clearance of remnant particles and increased plasma TG in humans.

## Patients and Methods

### Study design

68 healthy men and women of European descent were recruited by a newspaper advertisement and from our previous cohorts of healthy subjects. Inclusion criteria included age 18–65 years, nonsmoking, and no history of CVD or other severe disease. The subjects were screened to exclude any secondary condition affecting lipid levels or abnormalities in thyroid, liver, or kidney function or in basic blood count. Other exclusion criteria were use of medications affecting lipid or glucose metabolism, glycated hemoglobin A1C >6.4%, and fasting glucose >6.0 mmol/l. The study was approved by the Ethical Committee of Helsinki University Central Hospital. All subjects received written (and verbal) information and signed an informed consent. This form was approved by the ethics committee. All clinical investigation was conducted according to the principles expressed in the Declaration of Helsinki.

### Oral Fat Load

After a 12-h fast, subjects consumed a mixed meal consisting of bread, butter, cheese, skim milk, and tea or coffee (57 g fat, 63 g carbohydrates, and 40 g protein, total 934 calories) in the morning. Blood samples were drawn before and 3, 4, 6, and 8 hours after the meal. During this time, only water was served *ad libitum*. The subjects remained physically inactive throughout the study.

### Separation of lipoproteins and biochemical methods

Chylomicrons, VLDL_1_ and VLDL_2_ were isolated by gradient ultracentrifugation [21]. Concentrations of TG, cholesterol, apolipoprotein (apo) B48, and apoB100 were analyzed in plasma and in isolated lipoprotein fractions [21]. Plasma apoB48 was determined with an ELISA (AKHB48, Shibayagi, Japan). Plasma glucose was measured as described [21], and plasma insulin was assessed by ELISA (Mercodia, Uppsala, Sweden). After a heparin bolus of 70 IU/kg, blood samples were obtained, and LPL and hepatic lipase activities were determined as described [22].

### Determination of liver, subcutaneous, and visceral fat

Liver fat was measured by proton magnetic resonance spectroscopy and subcutaneous, abdominal, and visceral fat were measured by magnetic resonance imaging (MRI) [23]. Total abdominal fat was calculated as the sum of visceral and subcutaneous fat. 

### Genetic studies

HSPG synthesis is regulated by >35 genes, of which syndecan-1 (SDC1) and genes associated with initiation, sulfation or degradation of HSPG have all been associated with TRL uptake in vitro. In this study we tested if common allelic variations in SDC1 as well as in genes associated with HSPG initiation (N-acetylglucosaminyl-proteoglycan-4-beta-glucuronosyltransferase gene [also called exostosin-1 (*EXT1*)]), sulfation (heparin sulfate-2-O-sulfotransferase-1 gene: *HS2ST1*) or degradation (SULF1), were associated with disturbed hepatic clearance of TRL remnant particles. Single nucleotide polymorphisms (SNPs) rs2281279 in *SULF2*, rs10955854 in *EXT1*; rs1199668 in *HS2ST1*; and rs7607854 in *SDC1* were analyzed using Taqman PCR method (Applied Biosystems, Foster City, CA USA). ABI Prism Sequence Detection Systems ABI 7900HT was used for post-PCR allelic discrimination by measuring allele-specific fluorescence. Genotype distributions did not deviate from Hardy-Weinberg equilibrium and concordance rate of repeated genotyping was 100%.

### Association between rs2281279 genotype and SULF2 expression

*SULF2* mRNA expression was assessed in 211 liver biopsies of subjects of European descent, from the Advanced Study of Aortic Pathology biobank [24]. Gene expression was measured by the use of Affymetrix GeneChip Human Exon 1.0 ST arrays and DNA was genotyped by Illumina Human 610W-Quad Beadarrays as described [25].

### Statistical analysis

Values are presented as median and interquartile range or as number and proportion. The area under curve (AUC) and incremental area under curve (IAUC) for postprandial variables were calculated according to the trapezoid rule. Genotype and allele frequencies and categorical variables were compared by Fisher’s exact test or χ^2^ test. Differences in continuous variables between the genotypes were assessed by linear regression analysis after adjustment for age, gender, and body mass index. Non-normally distributed variables were log-transformed before entering the model. IBM SPSS Statistics version 20.0 was used for all statistical comparisons (IBM, Armonk, NY). 

## Results

### Study design

Oral fat load were performed in 68 healthy subjects (Table 1). The age of the study subjects was 47.3 ± 10 years (mean ± SD), and the distribution between genders male/females was 31/37. There were no significant differences between age and gender of the genetic variants analyzed (data not shown). Lipoproteins (chylomicrons, VLDL_1_, and VLDL_2_) were isolated and associations between responses of the postprandial lipids and common SNPs in genes encoding enzymes involved in the synthesis or degradation of HSPG were analyzed. Only rs2281279 in *SULF2* was associated with the postprandial response. The results for *EXT1*, *HS2ST1*, and *SDC1* are shown in Tables S1-S3. 

**Table 1:** Baseline Clinical Characteristics of Study Subjects.

No. of subjects	68
Age (years)	46 (41–53)
Male, n (%)	31 (46)
BMI (kg/m^2^)	24.1 (22.8–26.1)
Liver fat (%)	1.23 (0.57–3.49)
Total abdominal fat (cm^3^)	4366 (3315–5778)
Plasma TG (mmol/L)	0.84 (0.67–1.1)
Plasma cholesterol (mmol/L)	4.84 (4.31–5.19)
Plasma HDL-C (mmol/L)	1.58 (1.28–1.82)
Plasma LDL-C (mmol/L)	2.75 (2.46–3.19)
Plasma glucose (mmol/L)	5.40 (5.00–5.60)
Insulin	4.38 (3.24–6.02)
HOMA-IR	0.99 (0.78–1.50)
LPL activity (mU/mL)	174 (125–200)
HL activity (mU/mL)	170 (124–237)

Values are median (interquartile range) or *n* (%). Fasting triglycerides were measured at the oral fat load visit. HOMA-IR, homeostasis model assessment for insulin resistance

### Baseline Characteristics of the Study Subjects by the rs2281279 in SULF2

The frequency of rs2281279 minor G-allele was 0.17 and not different (*P*=0.77) from the frequency earlier reported for the Finnish population (www.1000genomes.org). Carriers of the G allele had lower fasting plasma TG, lower glucose, and higher LPL activity (*P*=0.037, 0.018, and 0.040, respectively; Table S4) as compared to AA genotype carriers. The rs2281279 did not associate with liver fat content nor with subcutaneous-, abdominal-, or visceral fat depots, or other anthropometric/metabolic variables (Table 1).

### Postprandial triglyceride rich lipoprotein metabolism

**Figure 1:**
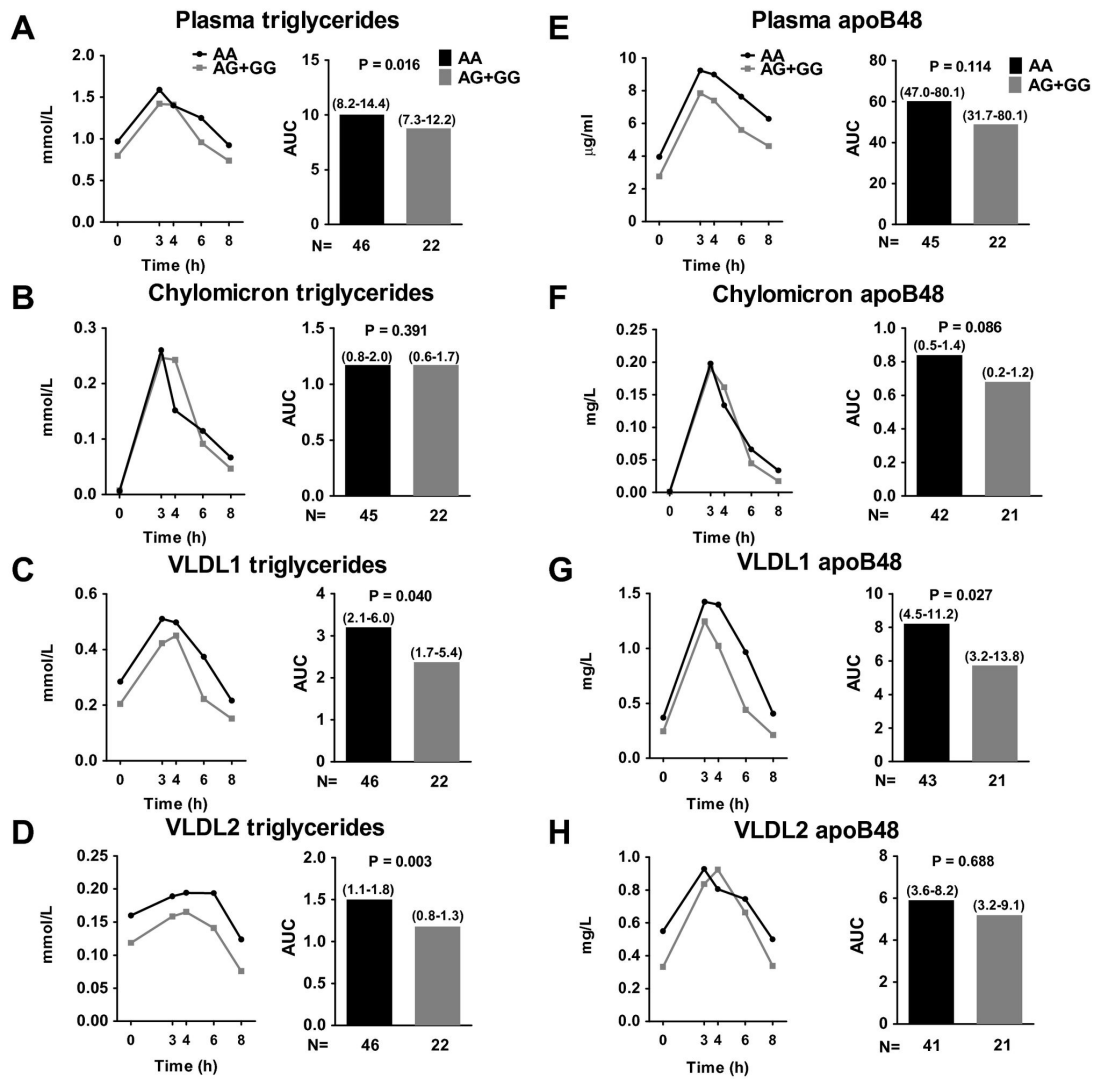
Postprandial TG and apoB48 in plasma and in lipoprotein subclasses stratified by SULF2 rs2281279 genotype. Carriers of the G allele had lower AUC for plasma TG (A) and TG content of VLDL1 (C) and VLDL2 (D) than AA homozygotes; the chylomicron TG content (B) did not differ between the different genotype carriers. Carriers of the G allele had lower AUC for apoB48 content of chylomicrons (F) and VLDL1 (G) than AA homozygotes; the apoB48 content in plasma (E) and VLDL2 (H) did not differ between the genotype groups. Data are shown as median (interquartile range). *P* values were calculated by linear regression adjusting for age, gender and body mass index (BMI). All the data is specified in Table S5. The number of subjects is given at the bottom of each column. AA, subjects with two A alleles; AG, heterozygotes; GG, subjects with two G alleles.

After an oral fat load, G allele carriers had a lower AUC for plasma TG (*P*=0.016, Figure 1A, Table S5). We next examined the relative TG content in isolated lipoprotein subclasses. G allele carriers had a reduced response (AUCs) for TG in the VLDL_1_ and VLDL_2_ fractions (i.e., TG in VLDL_1_ and VLDL_2_ particles and in remnant particles of similar densities) (*P*=0.040 and 0.003, respectively; see Figure 1C, 1D and Table S5). No differences were observed in the postprandial chylomicron TG content between the different rs2281279 genotype carriers (Figure 1B and Table S5).

Next, we measured the amount of intestinal and hepatic apoB isoforms (apoB48 and apoB100, respectively) in the lipoprotein subclasses. Carriers of the G allele had statistically significant reductions in apoB48-containing lipoproteins with VLDL_1_ density (i.e., chylomicron remnants within the VLDL_1_ density range) (Figure 1G, P=0.027) but not in the amount of apoB48 in VLDL_2_ (i.e., chylomicron remnants within the VLDL_2_ density range) or in nonlipolyzed chylomicrons as compared to AA-genotype carriers. Carriers of the G allele showed a nonsignificant trend toward lower apoB100 levels in VLDL_1_ and VLDL_2_ than carriers of AA-genotype (Table S5).

LPL activity correlated negatively with both fasting TG (*r*= –0.47, *P*<0.001) and the response (AUC) of postprandial plasma TG (*r*= –0.38, *P*<0.005). LPL also correlated negatively with AUCs of the TG content of chylomicrons (*r*= –0.26, *P*<0.05) and VLDL_1_ (*r*= –0.37, *P*<0.01).

### Association between rs2281279 genotype and SULF2 gene expression

We next analyzed mRNA expression levels in 211 liver biopsies from the Advanced Study of Aortic Pathology (ASAP) biobank. Allele and genotype frequencies of rs2281279 were in Hardy-Weinberg equilibrium (*P*=0.96 and 1.00, respectively). The minor allele frequency was 0.21 and not different from that of our study cohort (*P*=0.33). G allele of rs2281279 associated with lower *SULF2* mRNA expression in the liver biopsies (AA (N=131): 7.64±0.34; AG (N=71): 7.58±0.37; GG (N=9): 7.27±0.39, *P*=0.008).

## Discussion

We set up this study to test whether polymorphisms in genes responsible for HSPG assembly or disassembly associate with atherogenic dyslipoproteinemias in humans. Our results suggest that the SNP rs2281279 in *SULF2* associates with postprandial clearance of TRLs and with TG levels in healthy subjects, and that the reductions in fasting and postprandial TG levels are due to decreased postprandial TG content in the VLDL_1_ and VLDL_2_ fractions. These lipoprotein fractions contain chylomicron remnants, VLDL_1_, or VLDL_2_ and their VLDL remnants. Our results further support the finding that the major increase in postprandial lipoproteins occurs in VLDL remnant particles [7,8].

The fact that the turnover of chylomicron TG was similar in the two genotype groups indicates that the lipolysis of TRL particles is not associated with the *SULF2* polymorphism. This possibility is further supported by the significant negative correlations between LPL activity and the postprandial TG response of both chylomicrons and VLDL_1_. Consistent with this finding, carriers of the G allele had a reduced response of apoB48 in the VLDL_1_ fraction as compared to AA homozygotes, suggesting improved clearance of chylomicron remnant particles. A similar trend towards a reduced apoB100 response in both VLDL_1_ and VLDL_2_ was observed in G allele carriers, consistent with improved clearance of VLDL remnants. 

In healthy subjects, chylomicron remnants and VLDL remnants compete for clearance [26]. We have further shown that chylomicrons and VLDL particles are not cleared equally by LPL and that chylomicrons seem to be the preferred substrate [27]. Therefore, apoB48-containing remnants should be cleared preferentially, in line with our findings in this study. 

So far, data on the role of *SULF2* variants in humans is limited to an abstract report of subjects with type 2 diabetes, in whom *SULF2* rs2281279 was associated with the levels of both fasting and postprandial TRLs [28]. We extended that preliminary observation in an independent population by showing that *SULF2* variation also associates with fasting TG concentration and postprandial TRL levels in healthy normolipidemic subjects and that clearance of both chylomicron remnants and VLDL remnants may be modulated by *SULF2* genotype. Of note, the SNP rs2281279 in *SULF2* associates with hepatic *SULF2* mRNA levels. This result could imply that the *SULF2* rs2281279 SNP (or a variant in linkage disequilibrium with it) is involved in regulating *SULF2* expression.

The importance of the syndecan-1 HSPG pathway for hepatic clearance of TRLs in mice has been demonstrated by systemic deletion of syndecan-1 and hepatocyte-specific inactivation of sulfotransferases involved in HSPG biosynthesis [16,29,30]. Furthermore, treatment with SULF2 antisense oligonucleotides normalizes the defective clearance of TRLs that results from increased degradation of HSPGs by SULF-2 in animal models of diabetes [13,19,20]. 

 The strengths of this study lie in the design with a thorough phenotypic characterization combined with oral fat load analyses. However, we fully acknowledge that a significant drawback with this study design is that it limits the number of study subjects and consequently the statistical power to detect a genetic association. Further weaknesses are that we only genotyped one SNP per gene despite the fact that each gene has many common variants for European-descent populations, and that the multiple testing performed since p-values (significance) would not survive a conservative correction for multiple testing. It is therefore important to verify the results from this study in independent cohorts. Due to the small sample size, we combined the carriers with the G allele (AG+GG) when analyzing the associations between responses of the postprandial lipids and the SNP rs2281279 in *SULF2*. However, due to the larger study cohort, we were able to analyze the genotypes separately when analyzing the association between rs2281279 genotype and SULF2 gene expression. 

In summary, our results showing that mild, but clinically relevant, hyperlipidemias in human patients may result from genetic polymorphisms that affect hepatic HSPG; these findings indicate that novel targets that improve HSPG-mediated clearance of remnant particles may be an attractive strategy to improve metabolic dyslipidemia.

## Supporting Information

Table S1 **According to SCD1 rs7607854 Genotype.**
(PDF)

Table S2 **According to *EXT1* rs10955854 Genotype.**
(PDF)

Table S3 **According to *HS2ST1* rs1199668 Genotype.**
(PDF)

Table S4 **Baseline clinical characteristics according to *SULF2* rs2281279 Genotypes.**
(PDF)

Table S5 **According to *SULF2* rs2281279 Genotype.**
(PDF)
