# Supplementary material for: Genetic Variation in SULF2 Is Associated with Postprandial Clearance of Triglyceride-Rich Remnant Particles and Triglyceride Levels in Healthy Subjects
Source: PLoS One. 2013 Nov 20;8(11):e79473. doi: 10.1371/journal.pone.0079473 (PMC3835823; doi:10.1371/journal.pone.0079473)
Supplement: Table S1 — According to SCD1 rs7607854 Genotype. (PDF) [file pone.0079473.s001.pdf]

Genetic Variation in SULF2 Is Associated with Postprandial Clearance of Triglyceride-rich Remnant Particles and Triglyceride Levels in Healthy Subjects

Supplementary Table S1. According to *SDC1* rs7607854 Genotype

	**TT**		**TC**		
	Median	Interquartile range	Median	Interquartile range	*P*
No. of subjects, n (%)	57 (88)		8 (12)		
BMI (kg/m^2)	24.0	22.8–26.3	24.0	21.4–25.5	0.25
Plasma TG (mmol/L)	0.8	0.7–1.1	0.8	0.7–1.0	0.67
Plasma glucose (mmol/L)	5.4	5.0–5.6	5.2	4.9–5.8	0.71
Area Under the Curve					
Plasma-TG	9.5	8.1–14.0	8.6	6.8–12.0	0.073
Chylo-TG	1.2	0.8–1.7	1.4	0.5–2.2	0.94
$VLDL_1$-TG	3.1	2.1–6.0	2.4	1.3–3.4	0.023
$VLDL_2$-TG	1.3	1.0–1.7	1.00	0.9–1.7	0.18
Plasma apoB48	54.6	36.8–81.5	59.1	49.0–74.6	0.87
Chylo-apoB48	0.8	0.6–1.4	0.4	0.2–1.4	0.24
$VLDL_1$-apoB48	7.8	4.3–13.1	5.2	3.1–8.2	0.14
$VLDL_2$-apoB48	5.6	3.3–8.7	5.2	4.6–6.7	0.54
Chylo-apoB100	0.6	0.33–1.1	0.5	0.2–0.7	0.11
$VLDL_1$-apoB100	135.1	86.6–227.5	100.4	61.9–163.1	0.19
$VLDL_2$-apoB100	208.9	157.7–282.4	206.2	175.1–272.2	0.91

P values were calculated by linear regression analysis after adjustment for age, gender, and body mass index.

TT, subjects with two T alleles; TC, heterozygotes; Chylo, chylomicron
