# Supplementary material for: Genetic Variation in SULF2 Is Associated with Postprandial Clearance of Triglyceride-Rich Remnant Particles and Triglyceride Levels in Healthy Subjects
Source: PLoS One. 2013 Nov 20;8(11):e79473. doi: 10.1371/journal.pone.0079473 (PMC3835823; doi:10.1371/journal.pone.0079473)
Supplement: Table S2 — According to EXT1 rs10955854 Genotype. (PDF) [file pone.0079473.s002.pdf]

Genetic Variation in SULF2 Is Associated with Postprandial Clearance of Triglyceride-rich Remnant Particles and Triglyceride Levels in Healthy Subjects

Supplementary Table S2. According to *EXT1* rs10955854 Genotype

	TT		TG+GG		
	Median	Interquartile range	Median	Interquartile range	*P*
No. of subjects, n (%)	31 (46)		33+3 (54)		
BMI (kg/m^2)	23.8	22.6–25.7	25.1	23.0–26.4	0.36
Plasma TG (mmol/L)	0.8	0.6–1.0	0.9	0.7–1.3	0.08
Plasma glucose (mmol/L)	5.3	4.9–5.6	5.4	5.1–5.6	0.25
Area under the Curve					
Plasma-TG	8.8	7.7–10.7	11.3	8.1–14.9	0.24
Chylo-TG	1.0	0.7–1.5	1.3	0.7–2.1	0.30
$VLDL_1$-TG	2.6	1.7–3.7	3.5	2.1–6.7	0.34
$VLDL_2$-TG	1.2	1.0–1.6	1.5	1.0–1.8	0.17
Plasma apoB48	54.7	34.8–74.2	54.5	44.4–90.9	0.32
Chylo-apoB48	0.8	0.4–1.3	0.8	0.4–1.4	0.61
$VLDL_1$-apoB48	6.3	4.2–13.2	7.6	4.0–10.2	0.42
$VLDL_2$-apoB48	6.5	4.1–9.2	5.3	3.2–7.7	0.26
Chylo-apoB100	0.5	0.3–0.9	0.6	0.4–1.0	0.22
$VLDL_1$-apoB100	113.7	81.4–187.2	135.7	83.8–246.6	0.75
$VLDL_2$-apoB100	227.9	162.8–269.0	198.3	151.6–296.1	0.59

P values were calculated by linear regression analysis after adjustment for age, gender, and body mass index.

TT, subjects with two T alleles; TG, heterozygotes; GG, subjects with two G alleles; Chylo, chylomicron
