# Supplementary material for: Genetic Variation in SULF2 Is Associated with Postprandial Clearance of Triglyceride-Rich Remnant Particles and Triglyceride Levels in Healthy Subjects
Source: PLoS One. 2013 Nov 20;8(11):e79473. doi: 10.1371/journal.pone.0079473 (PMC3835823; doi:10.1371/journal.pone.0079473)
Supplement: Table S3 — According to HS2ST1 rs1199668 Genotype. (PDF) [file pone.0079473.s003.pdf]

Genetic Variation in SULF2 Is Associated with Postprandial Clearance of Triglyceride-rich Remnant Particles and Triglyceride Levels in Healthy Subjects

Supplementary Table S3. According to *HS2ST1* rs1199668 Genotype

	**AA**		**AG**		**GG**		*P*
	Median	Interquartile range	Median	Interquartile range	Median	Interquartile	(additive)
No. of subjects, n (%)	20 (29)		42 (62)		6 (9)		
BMI (kg/m^2)	25.1	22.2–26.3	23.7	22.7–25.8	25.1	23.8–28.4	0.97
Plasma TG (mmol/L)	0.9	0.7–1.2	0.8	0.7–1.1	0.8	0.6–1.3	0.39
Plasma glucose (mmol/L)	5.4	5.1–5.5	5.3	4.9–5.6	6.0	5.5–6.0	0.21
Area under the curve							
Plasma-TG	10.1	7.5–14.2	9.3	7.9–13.4	9.0	8.2–14.2	0.85
Chylo-TG	1.2	0.5–1.6	1.2	0.8–1.8	0.8	0.7–2.0	0.56
$VLDL_1$-TG	2.9	1.6–6.2	3.0	1.8–5.3	2.9	2.1–5.9	0.78
$VLDL_2$-TG	1.2	1.0–1.6	1.3	1.0–1.7	1.4	1.1–1.6	0.74
Plasma apoB48	50.9	38.1–79.7	9.9	34.4–77.4	60.0	47.3–109.4	0.57
Chylo-apoB48	0.7	0.3–1.5	0.9	0.4–1.4	0.7	0.6–0.8	0.33
$VLDL_1$-apoB48	7.5	3.9–9.2	7.0	4.1–14.7	7.7	5.2–13.3	0.32
$VLDL_2$-apoB48	5.2	3.1–7.1	5.5	3.6–9.3	7.5	5.3–8.8	0.19
Chylo-apoB100	0.6	0.4–0.9	0.53	0.3–1.1	0.6	0.4–0.7	0.55
$VLDL_1$-apoB100	135.1	68.3–204.9	118.5	82.2–224.7	117.8	105.3–221.1	0.59
$VLDL_2$-apoB100	199.7	98.5–260.5	224.3	158.4–290.6	224.5	186.2–294.2	0.40

P values were calculated by linear regression analysis after adjustment for age, gender, and body mass index.

AA, subjects with two A alleles; AG, heterozygotes.; GG, subjects with two G alleles; Chylo, chylomicron
