# Supplementary material for: Genetic Variation in SULF2 Is Associated with Postprandial Clearance of Triglyceride-Rich Remnant Particles and Triglyceride Levels in Healthy Subjects
Source: PLoS One. 2013 Nov 20;8(11):e79473. doi: 10.1371/journal.pone.0079473 (PMC3835823; doi:10.1371/journal.pone.0079473)
Supplement: Table S4 — Baseline clinical characteristics according to SULF2 rs2281279 Genotypes. (PDF) [file pone.0079473.s004.pdf]

Genetic Variation in SULF2 Is Associated with Postprandial Clearance of Triglyceride-rich Remnant Particles and Triglyceride Levels in Healthy Subjects

Supplementary Table S4. Baseline Clinical Characteristics According to *SULF2* rs2281279 Genotypes

	***SULF*2 rs2281279 alleles**		
	**AA**	**AG+GG**	***P***
No. of subjects (%)	46 (68)	21+1 (32)	
Age (years)	46 (39–53)	46 (38–52)	0.75
Male, n (%)	21 (46)	10 (45)	0.99
BMI (kg/m^2)	23.9 (22.4–26.0)	25.0 (23.2–26.6)	0.17
Liver fat (%)	0.92 (0.54–2.90)	1.23 (0.47–2.80)	0.96
Total abdominal fat (cm^3)	4268 (3405–5614)	5128 (3110–6685)	0.87
Plasma TG (mmol/L)	0.93 (0.67–1.26)	0.78 (0.66—0.88)	0.037
Plasma cholesterol (mmol/L)	4.86 (4.32–5.26)	4.71 (4.29–5.06)	0.32
Plasma HDL-C (mmol/L)	1.59 (1.25–1.82)	1.54 (1.41–1.86)	0.50
Plasma LDL-C (mmol/L)	2.78 (2.43–3.26)	2.62 (2.43–2.89)	0.29
Plasma glucose (mmol/L)	5.40 (5.08–5.63)	5.15 (4.88–5.45)	0.018
Insulin	4.35 (3.01–6.35)	4.37 (3.43–5.18)	0.86
HOMA-IR	0.98 (0.76–1.56)	0.91 (0.82–1.24)	0.79
LPL activity (mU/mL)	144 (115–199)	185 (158–203)	0.04
HL activity (mU/mL)	145 (117–230)	186 (142–292)	0.14

Values are median (interquartile range) or *n* (%). [[*P* values were calculated by linear regression analysis including body mass index in the model. Non-normally distributed variables were log-transformed before entering the model. Genotype distributions are in Hardy-Weinberg equilibrium ($P > 0.05$). Fasting triglycerides were measured at the oral fat load visit
