# Supplementary material for: Genetic Variation in SULF2 Is Associated with Postprandial Clearance of Triglyceride-Rich Remnant Particles and Triglyceride Levels in Healthy Subjects
Source: PLoS One. 2013 Nov 20;8(11):e79473. doi: 10.1371/journal.pone.0079473 (PMC3835823; doi:10.1371/journal.pone.0079473)
Supplement: Table S5 — According to SULF2 rs2281279 Genotype. (PDF) [file pone.0079473.s005.pdf]

Genetic Variation in SULF2 Is Associated with Postprandial Clearance of Triglyceride-rich Remnant Particles and Triglyceride Levels in Healthy Subjects

Supplementary Table S5. According to *SULF 2* rs2281279 Genotype

	**AA (*n* = 46)**		**AG + GG (*n* = 21+1)**		
Area Under the Curve	Median	Interquartile range	Median	Interquartile range	*P*
Serum TG	10.0	8.2–14.4	8.8	7.2–12.2	0.016
Chylomicron TG	1.2	0.75–2.0	1.2	0.56–1.7	0.39
$VLDL_1$ TG	3.2	2.1–6.0	2.4	1.7–5.4	0.040
$VLDL_2$ TG	1.5	1.1–1.8	1.2	0.84–1.2	0.003
Plasma apoB48	60.3	47.0–80.2	48.9	31.7–80.1	0.11
Chylo-apoB48	0.84	0.47–1.4	0.68	0.23–1.2	0.086
$VLDL_1$ apoB48	8.2	4.5–11.2	5.7	3.2–13.8	0.027
$VLDL_2$ apoB48	5.9	3.6–8.2	5.2	3.2–9.1	0.69
Chylo-apoB100	0.58	0.33–0.99	0.55	0.32–1.2	0.72
$VLDL_1$ apoB100	137.8	87.4–215.3	109.7	68.6–216.2	0.062
$VLDL_2$ apoB100	233.9	163.8–317.6	203.2	113.6–248.6	0.065

P values were calculated by linear regression analysis including age, gender, and body mass index in the model. Non-normally distributed variables were log-transformed before entering the model.

AA, subjects with two A alleles; AG, heterozygotes; Chylo, chylomicron.
